# Comparative genome analysis of *Burkholderia phytofirmans* PsJN reveals a wide spectrum of endophytic lifestyles based on interaction strategies with host plants

**DOI:** 10.3389/fpls.2013.00120

**Published:** 2013-04-30

**Authors:** Birgit Mitter, Alexandra Petric, Maria W. Shin, Patrick S. G. Chain, Lena Hauberg-Lotte, Barbara Reinhold-Hurek, Jerzy Nowak, Angela Sessitsch

**Affiliations:** ^1^Department of Health and Environment, Bioresources Unit, Austrian Institute of Technology GmbHTulln, Austria; ^2^Department of Energy, Joint Genome InstituteWalnut Creek, CA, USA; ^3^General Microbiology, University of BremenBremen, Germany; ^4^Department of Agriculture and Life Sciences, Virginia Polytechnic Institute and State UniversityBlacksburg, VA, USA

**Keywords:** *Burkholderia phytofirmans* PsJN, endophyte, plant-microbe interaction, comparative genomics, PGPR

## Abstract

*Burkholderia phytofirmans* PsJN is a naturally occurring plant-associated bacterial endophyte that effectively colonizes a wide range of plants and stimulates their growth and vitality. Here we analyze whole genomes, of PsJN and of eight other endophytic bacteria. This study illustrates that a wide spectrum of endophytic life styles exists. Although we postulate the existence of typical endophytic traits, no unique gene cluster could be exclusively linked to the endophytic lifestyle. Furthermore, our study revealed a high genetic diversity among bacterial endophytes as reflected in their genotypic and phenotypic features. *B. phytofirmans* PsJN is in many aspects outstanding among the selected endophytes. It has the biggest genome consisting of two chromosomes and one plasmid, well-equipped with genes for the degradation of complex organic compounds and detoxification, e.g., 24 glutathione-S-transferase (GST) genes. Furthermore, strain PsJN has a high number of cell surface signaling and secretion systems and harbors the 3-OH-PAME quorum-sensing system that coordinates the switch of free-living to the symbiotic lifestyle in the plant-pathogen *R. solanacearum*. The ability of *B. phytofirmans* PsJN to successfully colonize such a wide variety of plant species might be based on its large genome harboring a broad range of physiological functions.

## Introduction

The growing demand for alternatives to the use of agrochemicals in agricultural production has increased interest in harnessing benefits of plants' colonization by ameliorating microorganisms. Bacterial endophytes residing inside plants without harming their host (Wilson, 1995) have received particular attention as many of them support plant growth, and improve their health status and adaptation to changes in edaphic conditions (Ryan et al., 2007; Compant et al., 2010b).

For a long time healthy plants were considered to be free of bacteria (Compant et al., 2012). Although over the past few decades our understanding of the role of bacteria in the plant rhizosphere has dramatically advanced, we still have only a limited knowledge of bacterial traits determining internal colonization of host plants and their endophytic life. The rhizosphere is a nutrient-rich microbial hotspot and thus a highly competitive living environment. To gain a competitive advantage some of the rhizosphere bacteria penetrate plant organs and sustain both saprophytic and endophytic lifestyles (Hardoim et al., 2008). The identified determinants of this competitive ability include production of antimicrobial compounds, detoxification of reactive oxygen species (ROS), and plant secondary metabolites by anti-oxidative enzymes, ring-cleaving by dioxygenases, a presence of efflux pumps (Martinez et al., 2009; Barret et al., 2011), and/or efficient acquisition of nutrients facilitated by various membrane transporters and excreted siderophores (Loaces et al., 2011).

Rhizosphere bacteria can enter plants through tissue wounds, stomata and lenticels, germinating radicles [reviewed by Sturz et al. (2000)], penetration of root hair cells (Huang, 1986), ingress at emergence points of lateral roots, and/or the zone of their elongation and differentiation (Reinhold-Hurek et al., 1998). Production of cell wall-degrading enzymes (Huang, 1986; Quadt-Hallmann et al., 1997) such as endoglucanase (Reinhold-Hurek et al., 2006) is linked to the facilitation of penetration. To actively penetrate the cell wall bacteria need to be able to attach themselves to the root surface and move along the root to find suitable entry points. The root surface colonization is guided by plant-released compounds, i.e., root exudates, which serve as signals for chemotactic movement of bacteria. This is generally achieved by flagella and adhesion to plant cells via curli fibers and pili (Dörr et al., 1998).

During the transition from the host rhizosphere to the plant endosphere colonizing bacteria must have the capacity for quick adaptation to a highly different environment (i.e., pH, osmotic pressure, carbon source, availability of oxygen). They also have to overcome plant defense responses to the invasion, i.e., production of ROS causing stress to invading bacteria (Zeidler et al., 2004). Thus, bacterial ability to establish endophytic populations is likely to depend on the recognition of signal molecules [e.g., two-component systems or extracytoplasmatic function (ECF) sigma factors], mobility, penetration capability, and capacity for adjustment of metabolism and behavior. Once inside plants, endophytes either become localized at the entry point, or spread throughout the plant (Hurek et al., 1994; Hallmann et al., 1997) and colonize intercellular spaces (Patriquin and Döbereiner, 1978), vascular system (Hurek et al., 1994; Bell et al., 1995), or even penetrate cells. Motility aided by flagella (Buschart et al., 2012), twitching motility (Böhm et al., 2007), and the production of cell wall-degrading enzymes might be involved in the spreading throughout plant organs and tissues (Compant et al., 2010a). Endophytes colonize an ecological niche similar to that of phytopathogens (Hallmann et al., 1997) and host-plant/endophyte interactions are often considered mutualistic—the microorganisms gain nutrients and a protected niche to occupy, whereas the host benefits from bacterial activities resulting in plant growth promotion, improved nutrient uptake, increased stress tolerance, control of plant pathogens, and induction of systemic resistance (Sturz et al., 2000). These processes are triggered and/or regulated by the production of phytohormones, N_2_-fixation, P-solubilization, siderophore, and antibiotic production (Arshad and Frankenberger, 1997).

Our idea of an endophytic life cycle is based on the observation of many different species of endophytes. To obtain a better understanding of the characteristics determining endophytic colonization of plants and sustaining bacterial life within host plants we analyzed and compared genomes of selected sequenced bacteria (Krause et al., 2006; Fouts et al., 2008; Yan et al., 2008; Bertalan et al., 2009; Taghavi et al., 2009, 2010; Kaneko et al., 2010), with a particular focus on *Burkholderia phytofirmans* strain PsJN (Weilharter et al., 2011). These endophytes do not only differ in their genome size but also in their endophytic lifestyle; some have a very narrow host range and are exclusively found in plants whereas others colonize many different plants and are also good colonizers of other environments such as soil or the rhizosphere.

Our comparative analysis evidenced, that although all strains included in the analysis are prominent for conferring various plant beneficial effects, they seem to have different strategies regarding their life as endophyte and in the soil/rhizosphere environment and are accordingly equipped with different genetic set-ups. We concluded that the capacity to colonize plants endophytically cannot be reduced to few genetic traits and that different bacteria have evolved differently in their adaptation to the plant environment.

## Materials and methods

### Bacterial endophytes and their genomes

The following genomes were used for comparative genome analysis; the NCBI project ID numbers are given in brackets: *B. phytofirmans* PsJN [(Weilharter et al., 2011); 17463], *Azospirillum sp*. B510 [(Kaneko et al., 2010); 32551], *Klebsiella pneumoniae* 342 [(Fouts et al., 2008); 28471], *Methylobacterium populi* BJ001 (unpublished; 19559), *Pseudomonas putida* W619 [(Taghavi et al., 2009); 17053], *Pseudomonas stuzeri* A1501 [(Yan et al., 2008); 16817], *Enterobacter sp*. 638 [(Taghavi et al., 2010); 17461], *Azoarcus sp*. BH72 [(Krause et al., 2006); 13217], and *Gluconacetobacter diazotrophicus* Pa5 [(Bertalan et al., 2009); 21071].

### *B. phytofirmans* PsJN gene prediction, annotation, and analysis tools

The genome assembly was loaded into IMG/M-ER for gene predictions and annotation as described in the “Standard Operating Procedure for the Annotations of Genomes and Metagenomes submitted to the Integrated Microbial Genomes Expert Review (IMG-ER) System” document (http://img.jgi.doe.gov/er/doc/about_index.html. The *B. phytofirmans* PsJN genome sequence data were deposited in GenBank (http://www.ncbi.nlm.nih.gov/Genbank) under project accession CP001052, CP001053, and CP001054 (Weilharter et al., 2011). GC-profiling was done employing GC-Profile, a free web-based analysis tool (http://tubic.tju.edu.cn/GC-Profile/).

### Comparative genome analysis

Abundances of specific protein-encoding genes were compared for selected genomes by making use of IMG-ER (version 4.1 February 2013). Proteins were routinely identified by domains for specific functions (pfam or TIGRFam), by clusters of orthologous groups (COG) or in some cases [e.g., quorum sensing (QS) systems, nitrogenase, ACC deaminase, IAA production] by similarity search (BLASTP, e-value < 1e-5) with sequences of proteins for which the function has been experimentally proven to the IMG protein databases of the selected genomes.

## Results and discussion

### Genome architecture and plasmid pBPHYT01

The 8.2 Mbp genome of *B. phytofirmans* PsJN consists of two chromosomes and one plasmid (NC_010681.1, NC_010676.1, and NC_010679.1) encoding a total number of 7405 genes (Weilharter et al., 2011). Ninety-two percent (6807) of all CDSs in the genome of *B. phytofirmans* PsJN showed highest homology with *Betaproteobacteria* (Weilharter et al., 2011), and 4547 genes (61.4%) were most closely related to *B. xenovorans* LB400 (Chain et al., 2006), with over 3371 genes showing more than 90% sequence homology.

**Figure 1:**
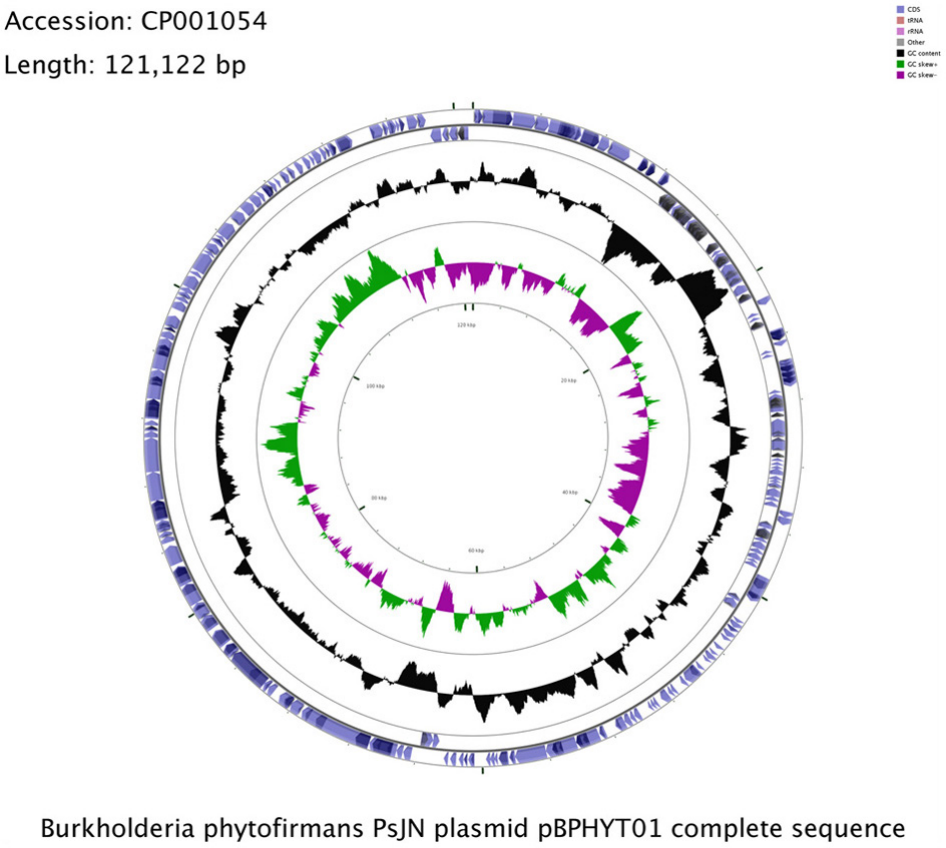
**Circular representation of the *Burkholderia pyhtofirmans* PsJN plasmid pBPHYT01.** The illustration is based on the visualization of sequence feature information by the GCView Server http://stothard.afns.ualberta.ca/cgview_server/ (Grant and Stothard, 2008).

*B. phytofirmans* PsJN harbors a plasmid of about 121 kbp (Figure 1), which is not homologous to the plasmids of *B. xenovorans* and *B. phymatum*, the closest relatives of strain PsJN. The G+C content of pBPHYT01 is 58%; lower than in the two chromosomes (63 and 62%, respectively). The plasmid sequence shows a uniform GC-profile (Gao and Zhang, 2006) and no distinct genetic islands. This indicates a unique origin of the plasmid rather than development through individual gene transfer events. An atlas of pBPHYT01 and information on the predicted origin of replication are publically available at the Genome Atlas Database (http://www.cbs.dtu.dk/services/GenomeAtlas-3.0/). We assume that the origin of replication is located around kbp 40.

Taghavi et al. (2010) identified distinct genetic islands for plant adhesion and colonization on the plasmid of *Enterobacter* sp. 638. No such regions linked to plant-associated lifestyle could be identified on the PsJN plasmid, pBPHYT01. Only 49 CDS (29%) out of 168 CDS located on pBPHYT01 could be functionally described; 119 CDS (71%) were annotated as hypothetical proteins. We identified a type IV secretion system, most probably involved in conjugal plasmid transfer, and several type II secretion system genes. It has been reported that pathogenic bacteria use type IV conjugation machineries to inject proteins into host cells [reviewed in Christie and Vogel (2000)]. For example, *B. cenocepacia* K56-2 excreted cytotoxic proteins involved in plant tissue water soaking are encoded on its plasmid's genes for the type IV secretion system (Engledow et al., 2004). On the other hand, type II secreted proteins are often involved in the degradation of plant cell wall components (Jha et al., 2005). The role of the plasmid-coded type II and type IV secretion systems in the endophytic lifestyle of *B. phytofirmans* PsJN remains ambiguous and requires further investigation.

### Architecture of endophyte genomes

**Figure 2:**
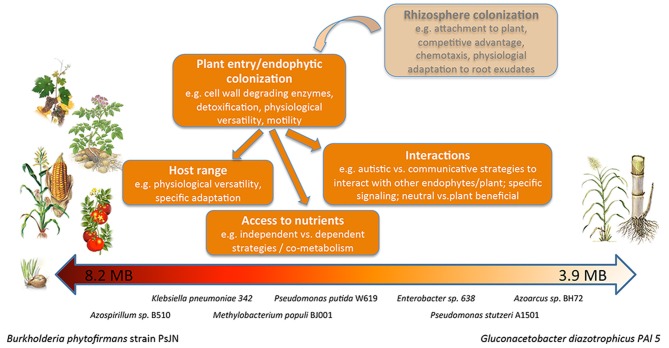
**The spectrum of endophytes analyzed in this study.** Drawing illustrating the differences in genome size and reported host spectrum of the endophytic bacteria analyzed in this study and summarizing the features of the endophytic lifestyle.

The genome sizes and genes content varies strongly in the nine studied endophytes, ranging from 3.9 Mbp and 3633 genes in *G. diazotrophicus* PAl5 and 4.4 Mbp and 3992 genes in *Azoarcus* sp. BH72, to 8.2 Mbp and 7487 genes in *B. phytofirmans* PsJN (Table 1, Figure 2). It is generally assumed that the genome size correlates with the number of possible lifestyles of a strain; a strain with higher gene content might be able to better deal with diverse environmental conditions. Toft and Andersson (2010) unveiled in their review that the genome size of bacteria drastically decreases during the evolutional transition from a free-living bacterium to an obligate intracellular symbiont. *B. phytofirmans* PsJN with its large genome is able to colonize various genetically unrelated plants such as potato and tomato (Conn et al., 1997; Nowak, 1998), grapevine (Compant et al., 2005), maize, switchgrass (Kim et al., 2012), sugarbeet, and barley (own unpublished data), both endophytically and in the rhizosphere. *B. phytofirmans* was first isolated from surface-sterilized onion roots (Frommel et al., 1991) and, in the description of the species *B. phytofirmans* (Sessitsch et al., 2005) was reported to occur in agricultural soils (Salles et al., 2006; Piccolo et al., 2010). In contrast, *Azoarcus* sp. BH72, characterized by a rather small genome size, to our knowledge has only been found in grasses and only reported as endophyte, not in soil (Reinhold-Hurek and Hurek, 1998) (Figure 1).

**Table 1:** **Architecture of endophyte genomes and reported habitats of the endophytes analyzed in this study**.

**Name**	***Bp*PsJN**	***Azo*B510**	***Kp*342**	***Mp*BJ001**	***Pp*W619**	***E*sp638**	***Pst*A1501**	***A*spBH72**	***Gd*PAl5**
Genome size	8.2	7.6	5.9	5.86	5.77	4.67	4.56	4.37	3.9
Genes	7487	6417	5425	5538	5292	4444	4146	3992	3633
NCBI taxon ID	398527	137722	507522	441620	390235	399742	379731	62928	272568
NCBI project ID	17463	32551	28471	19559	17053	17461	16817	13217	21071
CDS	7405	6309	5768	5464	5194	4274	4237	3989	3566
Coding bases	7119025	6707669	5219261	5085475	5182465	4149008	4123436	4024142	3557444
CRISPR count	0	7	1	0	0	2	1	0	4
rRNA	18	26	25	15	22	22	13	12	12
tRNA	63	79	88	58	75	84	61	56	55
Pseudo	164	0	0	99	12	35	9	0	65
Chromosomes	2	1	1	1	1	1	1	1	1
Plasmid	1	6	2	2	0	1	0	0	2
%GC	62	68	57	69	61	64	64	68	66
Reported habitats	Potato, tomato, grapevine, onion roots, maize, barley, agricultural soil	Rice	Maize, wheat	Poplar	Poplar	Poplar	Wheat	Kallar grass	Sugarcane, rice

### Mobile elements

**Figure 3:**
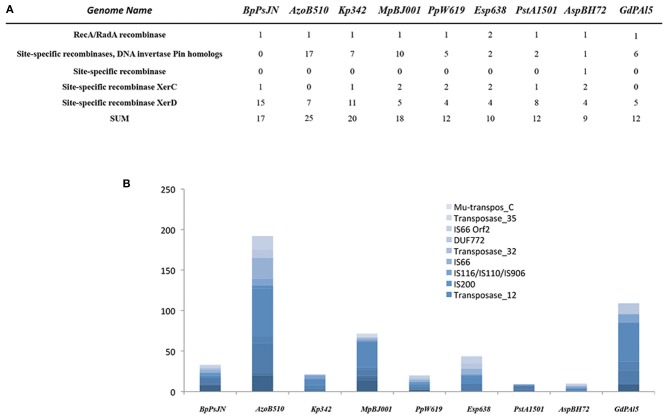
**Transposase genes (A) and mobile genetic elements (B) in the selected endophyte genomes**.

Mobile genetic elements play a significant role in bacteria–host adaption. Facultative symbionts contain 4–5 times more mobile DNA than obligate intracellular symbionts, reflecting a highly adaptive flexibility (Toft and Andersson, 2010). The endophytes analyzed in our study contain a rather low number of mobile elements (between 10 in *Enterobacter* sp. and 45 in *P. stutzeri* A1501), with the exception of *Azospirillum* sp. B510 (192), *G. diazotrophicus* PAl5 (109) and *M. populi* BJ001 (72) (Figure 3). Furthermore, only a few site-specific recombinases were encountered in their genomes; the gene content ranged from 9 in *Azoarcus* sp. to 25 in *Azospirillum* sp. B510 (Figure 3). This indicates high stability of their genomes, implying very good adaption to their habitats. However, in this context, only *Azoarcus* sp. BH72 can be considered as the most restricted endophyte, and its genome seemingly reflects its high habitat specificity. *Azospirillum* sp. B510 and *G. diazotrophicus* PAl5 are the only examples within the analyzed endophytes in which flexibility is likely to be achieved by a high number of mobile genetic elements.

### Motility and chemotaxis

Twitching motility refers to a flagella-independent form of bacterial movement over moist surfaces. It is mediated by the extension, tethering, and a following retraction of polar type IV pili. Twitching motility is important in host colonization by a wide range of plant and animal pathogens (Mattick, 2002) and crucial for endophytic colonization of rice by *Azoarcus* sp. BH72 (Böhm et al., 2007). We found genes for type IV pili in all bacterial genomes, except *P. stutzeri* A1501 (Table 2).

**Table 2:** **Functions relevant for rhizosphere competence and/or plant colonization in selected endophyte genomes**.

**Functions**		***Bp*PsJN**	***Azo*B510**	***Kp*342**	***Mp*BJ001**	***Pp*W619**	***E*sp638**	***Pst*A1501**	***A*spBH72**	***Gd*PAl5**
Motility and chemotaxis	Type IV pili	+	+	+	+	+	+	−	+	+
	Flagella	+	+	−	+	+	+	+	+	+
	Chemotaxis	+	+	−	+	+	+	+	+	+
	Methyl-accepting proteins	27	88	0	28	33	18	33	24	9
	Che protein response regulator	60	71	25	73	44	26	47	51	12
Plant adhesion	Curli fibers	−	−	−	2	5	5	7	−	−
	Hemagglutinin protein	1	−	2	−	1	1	−	−	−
	Agglutination protein	−	−	−	−	2	−	−	1	−
Plant polymer degradation	Glycoside hydrolases (GH) total	41	49	68	26	26	56	29	29	35
	Putatively plant polymer	14	17	22	12	11	16	18	12	8
	Degrading GH									
	% putatively plant polymer	34	35	32	46	42	28	62	41	23
	Degrading GH/GH									
Detoxification	Glutathione S-transferase	24	11	12	16	12	9	8	10	9
	Alkyl hydroperoxide reductase	2	1	1	0	1	1	2	1	1
	Thiol peroxidase	1	0	1	0	1	1	1	1	0
	Glutathione peroxidase	1	1	2	2	3	2	3	2	1
	Catalase/peroxidase	1	0	1	0	1	1	1	1	0
	Peroxidase	3	0	2	0	1	2	0	1	2
	SOD	2	2	3	2	3	3	2	2	1
	Catalases	6	2	4	6	4	5	2	2	3
	Peroxiredoxin	5	2	4	1	4	4	3	4	4
	SUM antioxidative enzymes	21	8	18	11	18	19	14	14	12
	Efflux pumps	681	597	554	275	361	385	223	232	209
Fe uptake	Dicitrate TonB-dependent receptor	4	2	4	8	6	3	6	9	16
	Linear catecholate TonB-dependent receptor	1	3	4	0	2	4	3	4	4
	Cyclic catecholate TonB-dependent receptor	0	0	0	1	1	0	0	0	0
	Hydroxymate TonB-dependent receptor	1	3	3	4	9	5	1	4	2
	SUM TonB-dependent receptors	6	8	11	13	18	12	10	17	22
	Siderophore biosynthesis	+	+	+	−	−	+	+	−	−
Degradation	Alkane monooxygenase	1		0	0	0	0	0	0	0
	Dioxygenases	15	16	9	1	11	0	8	15	0
Transporter	SUM transporter	1196	986	1082	617	830	801	613	524	510
	SUM transporter types	126	118	160	112	136	152	129	97	95
	No. of transporters/Mbp genome	146	130	183	105	144	172	134	120	131
	Porin	53	3	29	14	31	16	12	4	7
	ABC transporter	456	477	387	186	236	269	159	178	142
	Putrescin	+	+	+	+	+	+	+	−	−
	Spermidin/putrescin	−	+	+	−	−	+	−	−	−
Secretion systems	Type I	−	+	+	+	+	+	+	+	+
	Type II	+	−	−	+	+	+	+	+	−
	Type III	+	−	−	−	−	−	−	−	−
	Type IV	+	+	+	−	−	+	−	−	+
	Type Va	−	−	−	−	−	−	−	−	−
	Type Vb	−	−	−	−	+	−	−	−	−
	Type VI	+	+	+	−	+	−	−	+	−
Signaling	Two-component systems	272	n.d.	130	284	215	133	230	223	87
	Bacterial IQ	85	n.d.	65	115	n.d.	132	102	142	96
	ECF sigma factors	17	12	2	12	15	3	4	8	3
Quorum-sensing	3OH-PAME	+	−	−	−	−	−	−	−	−
	Autoinducer-2	−	−	+	−	−	+	−	−	−
	DSF-system	−	−	−	−	−	−	−	−	−
	AHL-based-system	+	+	−	+	−	−	−	−	+
	luxR-solo	+	+	+	−	+	+	−	−	−
	AHL-degradation	−	+	+	−	+	−	−	−	−
Plant growth promotion	N_2_ Fixation	−	+	+	−	−	−	+	+	+
	ACC deaminase	+	+	+	−	−	+	+	−	+
	IAA production	+	+	+	−	+	−	+	−	+
	

Genes involved in the biosyntheses of flagella formation are present in all endophytes except *K. pneumoniae* 342 (Table 2). The flagellum filament of invading bacteria is most likely one of the first structures to get in contact with plant cells and thus plants evolved mechanisms for its recognition that triggers their defense system. The lack of flagella in *K. pneumoniae* 342 might reflect adaption to the symbiotic lifestyle and might allow *K. pneumoniae* 342 to establish dense populations inside the host (Fouts et al., 2008). On the other hand, flagella are required for efficient endophytic colonization of rice roots by *Azoarcus* sp. BH72, where flagellins do not appear to act as PAMPS-eliciting defense responses (Buschart et al., 2012). Although we found a varying number of genes indicating chemotactic activity in all genomes, except that of *K. pneumoniae* 342, no correlation between gene content and genome size or host/habitat versatility could be determined (Table 2). As an example, *G. diazotrophicus* PAl5 has 12 genes for CheX proteins, transmembrane receptors, and two-component response regulators involved in the signal transduction for chemotaxis, whereas the equally small *Azoarcus* sp. BH72 has 51 such genes, a number similar to that of *B. phytofirmans* PsJN (60).

### Plant adhesion and root surface colonization

Successful plant colonization as well as the intensity, duration, and character of plant-microbe interactions depend on the bacterial ability to form adherent microbial populations (Danhorn and Fuqua, 2007). Bacteria usually interact with surfaces through adhesins such as polysaccharides and cell surface proteins. Curli are amyloid fibers involved in host cell adhesion and invasion as well as biofilm formation, cell aggregation, and immune system activation in many gram-negative bacteria (Barnhart and Chapman, 2006). Genes for curli fibers are present in *P. putida* W619 (5), *Enterobacter* sp. 638 (5) and *P. stutzeri* A1501 (7), and *M. populi* BJ001 (2) (Table 2). *P. putida* W619 and *Azoarcus* sp. BH72 carry genes for agglutination proteins. Hemagglutinin genes are present in *B. phytofirmans* PsJN (1), *K. pneumoniae* 342 (2), *P. putida* W619 (1), and *Enterobacter* sp. 638 (1). Hemagglutinins have formerly often been considered as “pathogenicity factors” as they were found to be important in both plant and human pathogenic bacteria (Balder et al., 2007; Gottig et al., 2009). Their widespread occurrence among bacterial endophytes indicates that hemagglutinin proteins may play crucial role in the invasion of eukaryotic cells by bacteria in general.

Interestingly, *Azospirillum* sp. B510 and *G. diazotrophicus* PAl5 apparently do not have genes involved in cell adhesion (Table 2) raising the question about how these bacteria get tightly adhered to plant cells. Type IV pili (Dörr et al., 1998) or flagella (Vande Broek et al., 1998) are proteins involved in adhesion to roots, and capsular material or exopolysacharides might as well-mediate attachment in these strains.

### Plant polymer-degrading enzymes

Many plant pathogens employ hydrolytic enzymes to soften and macerate plant cell wall polymers to release nutrients; a crucial component of phytopathogenic lifestyle leading to distraction of the host. However, hydrolytic enzymes such as glycoside hydrolases may also allow entry into, and translocation within the plant of both pathogens and non-pathogenic endophytes (Reinhold-Hurek et al., 2006). Glycoside hydrolases are very common in nature and besides the degradation of plant polymers their biological roles also include sugar metabolism, bacterial cell wall metabolism, and host-microbe interaction (Faure, 2002). In our survey we only selected those GH families, which may be putatively involved in plant polymer degradation (Table 2) (from http://www.cazy.org/Glycoside-Hydrolases.html). Corresponding GH genes were found in all endophytes, with *K. pneumoniae* 342 showing the highest GH gene content (22). When a number of putatively plant polymer-degrading GH genes was calculated in relation to the total number of GH genes in a given genome, we found *P. stutzeri* A1501 having the highest value (62%), followed by the *M. populi* BJ001, with 46%. *Enterobacter* sp. 638 (29%) and *G. diazotrophicus* PAl5 (22%) showed the lowest content of such genes. Moreover, we did not find any correlation between GH gene content, or diversity, and the reported range of host plants or microenvironments of the selected endophytes.

### Fe-uptake and siderophore production

Iron is essential to all living organisms as it serves as co-factor in various enzyme reactions. When iron availability is limited in the environment, bacteria capable of efficient iron acquisition have a competitive advantage irrespective of the environment, i.e., soil, the rhizosphere, or plant interior (Loaces et al., 2011). The plant microhabitat is poor in biologically available iron thus successful endophytes should be equipped with traits for its efficient acquisition. Many Gram-negative bacteria synthesize and excrete molecules with high affinity for iron, so called siderophores. Siderophores are either hydroxamate- or catechol-based and exist in different chemical variations (Miethke and Marahiel, 2007). The siderophores excreted into the extracellular environment bind iron and the uptake of ferric-siderophore complexes is achieved via ABC-type transporter proteins (TonB-dependent receptors) (Miethke and Marahiel, 2007).

Genes encoding TonB-dependent iron receptors were found in varying numbers in all selected endophyte genomes, ranging from 6 in *B. phytofirmans* PsJN to 22 in *G. diazotrophicus* PAl5 (Table 2). The high number of these genes in endophytes indicates that the root interior might be a particularly iron-depleted microenvironment (Reinhold-Hurek and Hurek, 2011), however, also other small molecules may be transported by TonB-dependent receptors. Siderophore production clusters were identified in only four strains; *B. phytofirmans* PsJN, *K. pneumoniae* 342, *Enterobacter* sp. 638 and *P. stutzeri* A1501. *B. phytofirmans* PsJN possesses a hydroxymate-type malleobactin siderophore biosynthesis cluster with over 70% amino acid identity to the malleobactin siderophore operon in *B. pseudomallei* K96243 (Alice et al., 2006).

Interestingly, those strains that do not produce siderophores encode a remarkably higher total number of outer membrane iron receptors than the siderophore producers (Table 2). It is known that bacteria often carry receptors for siderophores produced by other organisms (Cornelis and Bodilis, 2009). Therefore, we propose that two main strategies for iron acquisition might exist among endophytes—some bacteria are more independent, capable of the production and release of siderophores, whereas others depend on siderophores produced by other organisms and thus on a close interaction with other endophytes, or monocotyledonous host plants and fungi as they also may produce siderophores (Crowley et al., 1991).

Plants also benefit from the symbiosis with siderophore-producing endophytes resulting in better iron supply for their growth (Carrillo-Castañeda et al., 2002), although they may have to compete with non-siderophore producing bacteria inhabiting their interior.

### Detoxification

To survive in both environments, the rhizosphere and the plant interior, endophytes must be well-equipped with detoxification traits. Plant pathogens, and to certain extent also non-pathogenic microorganisms often elicit a defence response in plants including an oxidative burst (Buonaurio, 2008). Furthermore, plants are exposed to a range of abiotic or biotic stresses leading to the production of various ROS (including superoxide, hydrogen peroxide, and hydroxyl and hydroperoxyl radical species) and nitric oxide (Zeidler et al., 2004). It is therefore not surprising that numerous genes encoding for the detoxification of ROS such as catalases, superoxide dismutases, peroxidases, hydroperoxide reductases, and glutathione-S-transferases (GST) in all selected endopyhte genomes have been found in the studied endophytes (Table 2). An impressively high number of GST genes (24 copies) was found in the *B. phytofirmans* strain PsJN genome. GSTs are enzymes that detoxify endobiotic and xenobiotic compounds by covalent linking of glutathione to hydrophobic substrates (Vuilleumier and Pagni, 2002). Natural substrates of bacterial GSTs are mainly compounds resulting from oxidative damage to cell components, such as lipids, DNA hydroperoxides, and hydroxyalkenals (Vuilleumier, 1997). Apart from that, GST genes are often part of operons responsible for the degradation of aromatic compounds indicating their importance in these metabolic pathways (Vuilleumier, 1997). Comparison of the number of GSTs in *B. phytofirmans* PsJN with 60 other *Burkholderia* sp. genomes (available on the IMG/M-ER platform; http://img.jgi.doe.gov/er/doc/about_index.html) indicated that PsJN has an exceptionally high number of these genes even within its own genus (data not shown). *B. xenovorans* LB400 is the closest described relative of strain PsJN, which is known for its strong degradation capacity of aromatic hydrocarbons such as biphenyl or polychlorinated biphenyls (Rehmann and Daugulis, 2006), and possesses 18 GST genes. It has been reported that flavonoids and other polyphenols produced by plants inhibit the activity of rat liver GSTs in a concentration-dependent manner (Zhang and Nagaratnam, 1994). In this context we may speculate that the high number of GST-encoding genes of *B. phytofirmans* PsJN could confer its ability to cope with and make use of a broad spectrum of plant secondary metabolites and thus gives it an adaptive advantage.

Efflux pumps mediate the active transport of xenobiotics through the cytoplasmatic membrane that may play an important role in rhizosphere competence (Martinez et al., 2009; Barret et al., 2011). All selected endophytes encode hundreds of genes for efflux pumps (Table 2), with *B. phytofirmans* PsJN encoding the highest (681) and *G. diazotrophicus* PAl5 the lowest number of genes (201).

### Degradation of organic compounds

Plants produce complex organic compounds and it is thus expected that endophytes have evolved traits enabling them to degrade such metabolites. Our genome survey revealed that *B. phytofirmans* PsJN carries genes coding various degradation enzymes, such as alkane monooxygenase (*alkB*, Bphyt_5401) and cytochrome P450 alkane hydroxylase (Bphyt_1856). AlkB enzymes are required for break-down of aliphatic hydrocarbons. Aromatic compounds are usually oxidized by mono- or dioxygenases. At least 15 dioxygenase genes were found in the genome of *B. phytofirmans* PsJN, including a high number of ring cleavage enzymes such as catechol 1,2-dioxygenase, catechol 2,3-dioxygenase, 2-nitropropane dioxygenase, and protocatechuate 3,4-dioxygenase. Among the other selected endophytes only the genomes of *Azoarcus* sp. BH72 and *Azospirillum* sp. B510 indicate high degradation capacity, while *Enterobacter sp*. 638 and *G. diazotrophicus* PAl5 lack degradation genes (Table 2). The ability to degrade complex organic compounds is thus not common among endophytes. This is a surprising finding as the sequence analysis of the metagenome of the bacterial community in roots of field grown rice revealed a high number of genes encoding enzymes putatively degrading aliphatic and aromatic compounds leading to the assumption that this enzymatic capacity might be important for an endophytic lifestyle of bacteria (Sessitsch et al., 2012).

### Membrane transporters

Transport of nutrients and excretion of toxins are key cellular events, mediated by special transport proteins that mediate active and passive transport of solutes across the membrane. The number and composition of transporters depends on the genome size and the lifestyle of an organism (Gelfand and Rodionov, 2007). All selected endophyte genomes contain a high number of transporter genes ranging from 510 genes comprising 95 different transporter types in *G. diazotrophicus* PAI5, up to 1196 genes and 126 types in *B. phytofirmans* PsJN (Table 2). We calculated the transporter gene content in relation to the genome size of the endophyte and found that *M. populi* BJ001 contains the lowest relative number of transporter genes (105 genes/Mbp). The two *Enterobacteriaceae, K. pneumoniae* 342 and *Enterobacter* sp. 638 possess the highest number of transporter genes (183 and 172 genes/Mbp, respectively) and contain the richest transporter types with 160 and 152 different types in *K. pneumoniae* 342 *Enterobacter* sp. 638, respectively (Table 2).

Transport through membranes can occur either by energy-independent diffusion of solutes down a concentration gradient, or by energy-consuming active transport against a concentration gradient (Davidson et al., 2008). Energy-independent diffusion is mediated by porin channels, aqueous pores in the outer membrane of gram-negative bacteria allowing non-specific diffusion. Genes encoding porin channels (COG are present in all analyzed endophyte genomes. Interestingly strain PsJN possesses 53 porin genes, a high number in comparison to the other endophytes, which contain 29 or less porin genes (Table 2). This high number of porin channel-encoding genes is also observed in other *Burkholderia* species, and its close relative, *B. xenovorans* LB400, which possesses 91 porin genes. The energy-dependent transport system employs transporters that can be distinguished from each other based on their use of energy source (Davidson et al., 2008). The ABC superfamily is the largest group among the primary active transporters and the energy for transport is gained by the hydrolysis of ATP. The selected endophytes contain related genes ranging from 142 genes in *G. diazotrophicus* PAI5 to 477 genes in *Azospirillum* sp. B510 (Table 2). Interestingly, all endophytes but *Azoarcus* sp. BH72 and *G. diazotrophicus* PAI5 have putrescine transporters (Table 2). *Azospirillum* sp. B510, *K. pneumoniae* 342, and *Enterobacter* sp. 638 have in addition spermidine/putrescine transporters. Putrescine, 1,4-diaminobutane, is a biogenic polyamine present in almost all living cells (Igarashi and Kashiwagi, 2000). In bacteria hyperosmotic stress response includes the export of high amounts of putrescine. The osmolarity of rhizophere soil is expected to be higher than in bulk soil or plants. Thus, rapid adaption of bacteria to increased osmolarity may aid rhizosphere colonization (Miller and Wood, 1996). On the other hand putrescine content in plant cells and protoplasts increases dramatically upon osmotic stress (Flores and Galston, 1982). Kuiper et al. (2001) demonstrated that increased uptake of putrescine in the rhizosphere inhibits root colonization by *Pseudomonas fluorescens* WCS365. We may speculate, whether the lack of intact putrescine transporters in *Azoarcus* sp. BH72 isolated from roots of salt-stressed plants grown in saline-sodic soil and *G. diazotrophicus* PAI5 might reflect a strategy of protection from plant-produced putrescine and adaption to the plant habitat.

### Secretion systems

The secretion of proteins plays a central role in biotic interactions of bacteria. Up to now six types of protein secretion systems have been described for Gram-negative bacteria (Tseng et al., 2009). Proteins are either translocated across the inner and outer membrane in a single step (type I, type III, type IV, and type VI), or first transported into the periplasmatic space by universal Sec and two-arginine pathways and then exported mainly via the type II and type V secretion systems (T2SS, T5SS). Type III, type IV, and type VI secretion systems involve a translocation unit—kind of a needle—that allows the direct injection of proteins into the cytoplasm of host cells (Veenendaal et al., 2007). The importance of protein secretion systems in pathogenic as well as beneficial plant-microbe interactions, particularly that of T3SS, T4SS, and T6SS has been reported (Tseng et al., 2009). Many proteins (toxins or effector proteins) secreted by pathogens or symbionts have the ability to trigger defense responses or manipulate host cell structure and physiology supporting colonization, nutrition, and proliferation of the bacteria (Torto-Alalibo et al., 2009).

In general, we found all types of secretion systems in the analyzed endopyhtic genomes (Table 2), with T1SS being present in eight strains and T2SS in six strains. *B. phytofirmans* PsJN harbors at least four different secretion systems (T2SS, T3SS, T4SS, and T6SS), more than the other strains. The presence of T1SS in *B. phytofirmans* PsJN remains unclear. We found only one T1SS gene, namely that for the membrane fusion protein HlyD but did not find an inner- or outer membrane transport component. Strain PsJN is the only endophyte that possesses a T3SS. Reinhold-Hurek and Hurek (2011) also found the T3SS to be extremely rare among endophytes as they encountered T3SS genes only in *Herbaspirillum seropedicae* SmR1, an endophytic strain colonizing intracellular spaces in grasses (Pedrosa et al., 2011). This led to the suggestion that T3SS-based mechanisms for modulating plant response used by pathogens and some symbionts are not common in plant-endophyte interactions (Reinhold-Hurek and Hurek, 2011). Interestingly, *B. phytofirmans* PsJN has all relevant T3SS genes, but the gene for the needle-forming protein seems to be missing. As suggested previously (Reinhold-Hurek and Hurek, 2011), the T6SS appears to be more common among endophytes and was also abundantly represented in the metagenome of rice root endophytes (Sessitsch et al., 2012), indicating that it might play a role in the host-microbe interaction.

### Signaling

In order to sense and react to the extracellular environmental signals, cells must be able to transmit the information from the cell surface to the cytoplasm (i.e., the site of gene regulation). The two transmembrane signal-transduction mechanisms most commonly found in bacteria are two-component systems typically formed by a membrane protein with extracellular and intracytoplasmic domains and a soluble intracellular response regulator, or extracytoplasmic function (ECF) sigma factors. Both are mechanisms of coordinated cytoplasmic transcriptional regulation in response to signals perceived by protein domains external to the cell membrane.

### Two-component systems

Bacteria usually contain tens to hundreds of two-component systems controlling vital processes such as metabolism, development, motility, response to stress, or virulence. The complexity of the signaling systems correlates with the genome size, the phylogeny, the ecology, and metabolic properties of bacteria (Galperin, 2005). Galperin et al. (2010) introduced the term “bacterial IQ” referring to the total number of signaling proteins in a given proteome that can be used as a measure of a bacterium's ability to adapt to diverse environmental conditions. The information on signaling genes in the genome of the selected endophytes, except that of *Azospirillum* sp. B510 and *P. putida* W619, is publically available (http://www.ncbi.nlm.nih.gov/Complete_Genomes/SignalCensus.html). The total number of signaling genes range from 85 in *G. diazotrophicus* PAI5, up to 241 in *B. phytofirmans* PsJN (Table 2). *Azoarcus* sp. BH72 has a bacterial IQ of 142, the highest value among the selected endophytes. Interestingly, for the huge genome of *B. phytofirmans* PsJN a bacterial IQ of “only” 85 has been calculated. This reflects most probably the phyologenetic background of strain PsJN. The bacterial IQ values of the *Burkholderia* species range between 57 in *B. xenovorans* and 93 in *B. multivorans* (Galperin et al., 2010).

### Extracytoplasmatic (ECF) function sigma factors

The ECF subfamily is the largest group among the σ70 family and its members are involved in a wide range of environmental responses, such as metal homeostasis, starvation, and resistance to antimicrobial peptides, being also required for pathogenesis in some cases (Helman, 2002). Recently, Gourion et al. (2009) showed that an extracytoplasmatic sigma factor is involved in stress response and symbiotic efficiency in the plant symbiont *Bradyrhizobium japonicum* USDA110. Knock-out mutants had defects in heat shock and desiccation resistance upon carbon starvation. Additionally, they induced fewer and smaller nodules on soybean and mungbean than the wild type and their specific nitrogenase activity was drastically reduced the first weeks after inoculation.

In general, the number of ECF sigma factors increases with genome size and the complexity of lifestyle (Helman, 2002); bacterial species living in different habitats encode more ECF sigma factors than bacteria that live in stable niches (Cases and de Lorenzo, 2005; Gourion et al., 2009). For example, plant pathogenic *Pseudomonas syringae* pathovars have much fewer ECF sigma factors than related pseudomonads with more complex lifestyles; *P. syringae* has 2, whereas the related species *P. aeruginosa* PAO1 and *P. putida* KT2440 contain 19 ECF sigma factors (Oguiza et al., 2005).

ECF sigma factor genes were found in all selected endophyte genomes with numbers ranging from 2 in *K. pneumoniae* 342 to 17 in *B. phytofirmans* PsJN (Table 2). Based on the current information on the possible habitats of the selected endophyte species we do not see a correlation between lifestyle (exclusively in plants or in both soil and plant) and the number of ECF sigma factors.

### Quorum sensing

QS is a regulatory mechanism used by bacteria to regulate gene expression in a cell density-dependent manner (Camilli and Bassler, 2006). This is achieved by sensing the local concentration of small molecules produced by the bacteria. QS-signal molecules regulate various functions essential for the successful establishment of pathogenic or symbiotic relationships (Loh et al., 2002). With the exception of *P. stutzeri* A1501 all analyzed endophyte genomes contain QS-related genes (Table 2). The autoinducer-2 system was identified in *K. pneumoniae* 342 and *Enterobacter* sp. 638, whereas N-acyl homoserine lactone (AHL)-based systems were detected in *B. phytofirmans* PsJN, *M. populi* BJ001, and *G. diazotrophicus* PAl5.

*B. phytofirmans* PsJN possesses genes for two LuxI and LuxR-type protein operons on chromosome 1 (Bphyt_0126/0127) and on chromosome 2 (Bphyt_4275/4277), and the production of 4 compounds (3-hydroxy-C8-homoserine lactones, 3-hydroxy-C14-homoserine lactones, 3-oxo-C12-homoserine lactones, and 3-oxo-C14-homoserine lactones was shown (Sessitsch et al., 2005; Trognitz et al., 2008). Recently, Zuñiga et al. (2013) reported that 3-hydroxy-C8-homoserine lactones play a role in swimming motility and are required for efficient colonization of *Arabidopsis thaliana*.

An additional LuxR-type regulator gene was discovered on chromosome 2 (Bphyt_6042), of *B. phytofirmans* PsJN which is not paired with a *lux*I gene. Such unpaired luxR-type genes are also present in the genomes of *Enterobacter* sp. 638, *K. pneumoniae* 342, and *P. putida* W619. *Azoarcus* sp. BH72 harbors a *luxI*-type synthesis protein, but until now no function was stated for this conserved hypothetical protein and it is known that it does not produce AHLs and cannot communicate through an AHL-based system (Krause et al., 2006). However, it could be clearly shown that this endophyte communicates via a so far unknown hydrophilic signal factor (Hauberg-Lotte et al., 2012). LuxR-*solo* proteins (Subramoni and Venturi, 2009) are not unusual and have been found in bacteria possessing one or several AHL-QS circuits, as well as in bacteria that do not have any LuxI synthases (Case et al., 2008). LuxR-*solo* proteins recognize the AHLs produced by the resident QS system(s) and play a role in the regulation and balancing of the QS network (Wilkinson et al., 2002; Fuqua, 2006; Hoang et al., 2008). Moreover, LuxR-*solo* proteins might allow bacteria to sense and respond to AHLs produced by other species (Subramoni and Venturi, 2009). The unpaired LuxR genes in *Enterobacter* sp. 638, *K. pneumoniae* 342 and *P. putida* W619 are homologous to SdiA, an AHL receptor in *Salmonella enterica* serovar Typhimurium that exclusively detects signal molecules of other species (Michael et al., 2001). In this context it is interesting that various plant species including rice, pea, soybean, or tomato have been shown to produce and excrete substances mimicking AHL signal molecules and interfering with the QS system in bacteria (Bauer and Mathesius, 2004). Furthermore, it has been shown that luxR of plant-associated bacteria may sense plant compounds (Subramoni et al., 2011). It therefore might be that some endophytes make use of LuxR-*solo* proteins to sense and respond to the conditions inside the host plant, but luxR generally might also facilitate communication among endophytes.

Surprisingly all three important components for the 3-OH-PAME system were found in the genome of *B. phytofirmans* PsJN. They are organized in one cluster on chromosome 1 (Bphyt_1287/1288/1289). The 3-OH-PAME system has been described for *Ralstonia solanacearum* (Flavier et al., 1997), but it has not been reported for *Burkholderia* spp. so far. The signal molecule in *R. solanacearum* is a 3-hydroxypalmitic acid methyl ester (3-OH-PAME), which is synthesized by a S-adenosyl methionine-dependent methyl transferase (PhcB) that converts 3-hydroxypalmitic acid into a methyl ester (Flavier et al., 1997). At low 3-OH-PAME concentrations a periplasmatic two-component histidine kinase phosphorylates the response regulator PhcR, which in turn down-regulates the activity of the master regulator protein PhcA post-transcriptionally. PhcA up-regulates the expression of extracellular polysaccharides (EPS) and endonucleases, whereas at the same time it represses motility and siderophore synthesis (Clough et al., 1997; Garg et al., 2000). It was proposed that the Phc regulatory network serves as a key to mediate the switch in behavior needed when bacteria change from free-living to host-associated (von Bodman et al., 2003). Similar to *B. phytofirmans* PsJN *R. solanacearum* encodes two pairs of *lux*I/*lux*R homologs. The *so*lI/*sol*R operon has been described in detail and was found to be under control of PhcA (Flavier et al., 1997). Disruption of *so*lI/*sol*R did not influence virulence, EPS-, and exoenzyme production in *R. solanacearum* (Flavier et al., 1997). *B. phytofirmans* PsJN possesses all genes of the pheBSRQ operon that regulates phcA in *Ralstonia* sp. but lacks a gene homologous to the master regulator gene *phc*A. Apart from *B. phytofirmans* PsJN and *R. solanacearum* the 3-OH-PAME system is present in *R. pickettii*, some strains of the *Cupriavidus* genus and *Burkholderia* such as *B. xenovorans* LB400—all together belonging to the family *Burkholderiaceae*.

But why do bacteria make use of multiple QS-systems employing chemically different signal molecules? A reasonable explanation was given by Horswill et al. (2007). In some environments such as soil, bacterial cells are not always linked through the liquid phase and thus it might be a disadvantage to rely on a QS-system acting in the liquid phase. Volatile signaling would be favorable. Interestingly, 3-OH-PAME can act in the gas phase (Horswill et al., 2007). Apart from that, many bacteria are able to use AHLs as carbon and nitrogen source and thus might interfere with the QS system in other species (Horswill et al., 2007). So, the use of multiple, chemically different signals could stabilize the QS regulatory network against environmental perturbations. In this context, the presence of three QS-systems in *B. phytofirmans* PsJN might indicate its ability to successfully establish under diverse environmental conditions.

### Plant growth promoting functions

The proposed mechanisms of bacterial phytostimulation are linked to the direct production/modulation of plant hormones such as indoleacetic acid, gibberellic acid, cytokinins, and ethylene (Arshad and Frankenberger, 1997), and bio-fertilization through nitrogen fixation and enhancement of the uptake of mineral nutrients (Babalola, 2010; Hayat et al., 2011). Our survey of the genomes confirmed the presence of relevant genes within the selected genomes (Table 2) that have been previously reported elsewhere (Iniguez et al., 2004; Krause et al., 2006; Fouts et al., 2008; Bertalan et al., 2009; Taghavi et al., 2009; Kaneko et al., 2010; Weilharter et al., 2011).

## Conclusions

Based on comparative analyses of eight genomes of bacterial endophytes we addressed the question of occurrence of the overall features typical for, and required for the establishment of endophytic lifestyles by bacteria. Although our current idea of the endophytic lifestyle of bacteria is based on studies of a limited number of strains comprising only *Proteobacteria*, we found some common characteristics shared by all analyzed genomes, such as capability to overcome plant defenses. The genomic comparison revealed a high genetic diversity indicative of the phylogenetic groups the analyzed strains belong to. Characteristics such as motility, chemotaxis, and degradation of plant polymers and organic compounds have been proposed to be necessary for colonization and endophytic life (Reinhold-Hurek et al., 2006; Böhm et al., 2007; Hardoim et al., 2008). However, our study suggests that not all these traits are absolutely necessary. One of the surveyed endophytes, *K. pneumoniae* 342, does not have genes involved in biosynthesis of flagella, the feature that might suggest adaption to the symbiotic life via establishing dense populations inside the host (Fouts et al., 2008). The traits generally encountered in endophyte genomes include detoxification of ROS, a strategy to deal with plant defense responses similar to plant pathogenic bacteria (Lamb and Dixon, 1997), as well as the production of plant polymer-degrading enzymes. Similarly, quorum-sensing, an important trait in pathogens' invasion of plants is also prominent in the endophyte genomes, but not the presence of the type III secretion system.

Most of the analyzed endophytes, except *G. diazotrophicus* PAl5 and *M. populi* BJ001, carry only a low number of mobile elements and have relatively stable genomes, indicating that they employ other mechanisms than horizontal gene transfer to adapt to altering conditions. *G. diazotrophicus* PAl5 is a particularly interesting example as it has a small genome and lower genomic capacity for adaptation in comparison to the strains with larger genomes. Generally, facultative endophytes, i.e., endophytes, which are likely to live also in other environments (Hardoim et al., 2008), do not seem to have reduced genomes such as other symbionts. In contrast, large genomes and physiological variability might be an advantage for a facultative endophyte.

In several aspects different endophytes seem to have highly different life strategies. One example is the evolution of different strategies for the acquisition of iron. Four of the surveyed endophytes (*B. phytofirmans* PsJN, *K. pneumoniae* 342, *Enterobacter* sp. 638, and *P. stutzeri* A1501) evolved an independent acquisition system—they are able to produce, excrete, and bind siderophores sequestering iron and uptake it from the Fe-siderophore complexes. On the contrary, other endophytes fully depend on other organisms, which produce siderophores (Alice et al., 2006). This group is also characterized by a large number of outer membrane iron receptors capable of binding Fe-siderophores. The siderophore producers group is better aligned with the plant nutrition requirements as plants are able to access iron from the Fe-siderophore complexes (Crowley et al., 1991). Conversely, endophytes with a large number of iron receptors may compete with plants for iron acquisition, but likely act as biocontrol agents against fungal and bacterial pathogens.

Endophytes have evolved different communication strategies. Some strains have various QS strategies facilitating cell-to-cell communication between cells of the same strain and may use the signal compounds involved also to interact with other strains, potentially belonging also to other taxa. Some genes have been found such as luxR-*solos*, which are likely to respond to signal compounds of other endophytes or plants. It also has to be noted that QS signals such as AHLs may induce different types of plant responses (Hartmann and Schikora, 2012). In contrast, other endophytes lack such systems or possess yet unknown QS capabilities.

Based on the limited set of the analyzed genomes we also conclude that the degradation of complex organic compounds as carbon source, and/or during the detoxification process, is not common among endophytes. The finding that *B. phytofirmans* PsJN has the capacity for degradation of various hydrocarbons might be more related to the fact that members of the genus *Burkholderia* are generally well-equipped with the degradation pathways, including the degradation of photosynthates (Rasche et al., 2009). Nevertheless, degradation of various types of complex organic compounds may help strain PsJN to colonize different plant environments, including a broad spectrum of plant species (Compant et al., 2008b), their organs and tissues (Compant et al., 2008a). This hypothesis is also supported by the presence of numerous degradation genes potentially involved in the degradation of aromatic, aliphatic, and halogenated substrates in the rice endophyte metagenome (Sessitsch et al., 2012). A biogenic polyamine putrescine (Igarashi and Kashiwagi, 2000), one of the signaling molecules involved in plant and microbial adaptation to stresses, might also aid rhizosphere colonization (Miller and Wood, 1996). Two strains, *Azoarcus sp*. BH72 and *G. diazotrophicus* PAl5 with rather small genomes lack complete putrescine transporters. This indicates a high level of adaption to the plant interior as an exclusive habitat. To the best of our knowledge, both of these bacterial species have only been found in plants, not in soil or other habitats; with the exception of one related strain, *Azoarcus* sp. DS30, isolated from hexachlorocyclohexane-contaminated soil in India (Dadhwal et al., 2009).

Altogether, bacteria have evolved different strategies for colonization of plant organs and establishment *in planta*. The surveyed endophytes colonize completely different plant species (Table 1). *Azospirillum* sp. B510, *G. diazotrophicus* PAI5, *Azoarcus* sp. BH72, and *K. pneumoniae* 342 fix nitrogen and have been found only in grasses, whereas non-diazotrophic *M. populi* BJ001, *P. putida* W619 and *Enterobacter sp*. 638 colonize poplar trees, and *B. phytofirmans* PsJN is able to establish endphytic populations in a wide variety of plants including herbaceous and woody species.

Overall, plant species are highly diverse and differ strongly in their morphology, physiology, and biochemistry. There is a tendency to generalize the “plant habitat,” but the conditions and challenges bacteria are facing during their endophytic lifespan might be completely different in annuals, as compared to trees and herbaceous perennials.

*B. phytofirmans* PsJN is in many aspects a unique organism among the endophytes selected for this study. It has the far biggest genome consisting of two chromosomes and one plasmid, well-equipped with genes coding for degradation pathways of complex organic compounds, including detoxification involving GST. Furthermore, strain PsJN harbors a high number of cell surface signaling and secretion systems that allow it to interact with a variety of host plant species and adapt to different environments.

### Conflict of interest statement

The authors declare that the research was conducted in the absence of any commercial or financial relationships that could be construed as a potential conflict of interest.
